# The Analysis of Lead Phytotoxicity in Seeds Using CO_2_ Laser Photoacoustic Spectroscopy

**DOI:** 10.3390/molecules25071637

**Published:** 2020-04-02

**Authors:** Cristina Popa, Ana Maria Bratu, Mioara Petrus, Mihaela Bacalum

**Affiliations:** 1 National Institute for Laser, Plasma and Radiation Physic, Laser Department, 409 Atomistilor St., P.O. Box MG-36, Magurele 077125, Romania; ana.magureanu@inflpr.ro (A.M.B.); mioara.petrus@inflpr.ro (M.P.); 2 Horia Hulubei National Institute for R&D in Physics and Nuclear Engineering, Department of Life and Environmental Sciences (DFVM), Magurele 077125, Romania; bmihaela@nipne.ro

**Keywords:** analysis of gases, fluorescence microscopy, gas molecules, IR spectroscopy, plant respiration measurement

## Abstract

Lead (Pb) is the most prevalent heavy metal pollutant in the natural environment. Pb is not a fundamental element for plants, but they absorb it when it is present in their environment, having no known physiological activity. The aim of our research was to evaluate the efficacy of laser photoacoustic spectroscopy as a tool to monitor changes induced by Pb in plant respiration by highlighting two molecular markers (C_2_H_4_ and CO_2_). To better understand Pb phytotoxicity, we monitored the plantlets evolution as well as the morphology of the root cells. Firstly, we showed that the treatment hinders the plantlet’s development. Furthermore, using laser photoacoustic spectroscopy, we found a decrease in the concentration of C_2_H_4_ and CO_2_ vapors measured in the respiration of treated plants. Finally, fluorescence microscopy results showed that in Pb treated plantlets, the cell roots morphology is clearly altered as compared with the untreated ones. All the results are well correlated and can help further in understanding Pb phytotoxicity.

## 1. Introduction

Toxic metals are conventionally characterized by an atomic number >20, of which Cd, Cr, Cu, Hg, Pb, and Zn are the most common ones [1]. Some of these metals, such as Zn, Cu, Mn, Ni, and Co, can be found as natural components in soil and are micronutrients necessary for plant growth, whereas Cd, Pb, and Hg have unknown biological function [1,2]. Similar to all the metals, lead can be toxic to living organisms ranging from animals, plants, even to microorganisms, with effects mostly limited, particularly to contaminated areas [3,4,5,6].

As reported previously, the toxic metals pose a serious health problem also to humans, namely, by inducing brain damage and retardation [4]. Pb^2+^ was reported to be toxic to humans if it enters in high amounts in the body. Because Pb^2+^ is not biodegradable, if is found as a contaminant in the soil, it remains a long-term source of pollution, with destructive effects on all biological systems and environment [4].

Plants are also affected by lead accumulation. Although Pb can accumulate in different parts of the cells, previous studies showed that it is predominantly found in the root of the plants [7]. Pb accumulation leads to undesired changes in physiological processes, like: Plant growth, root elongation, enzyme activities, and photosynthesis, predominantly inhibiting them [8]. Aside from these, Pb was shown to alter membrane organization and functions [4,7,8]. Lead also causes an increase in reactive oxygen species (ROS) generation [9]. All these physiological changes are expected to also affect the volatile organic compounds generated by the affected plants.

Over the years, different methods were used to identify volatile organic compounds created by living organisms found under various stress conditions. Methods such as gas chromatography-mass spectrometry, infrared spectroscopy, or chemiluminescence show some limitations due to its low limit of detection, but also due to the complex protocols employed [10]. Photoacoustic spectroscopy proved to be a promising alternative to these methods, mostly due to its high sensitivity and selectivity, and also simple and reproducible protocols [10,11]. CO_2_ laser photoacoustic spectroscopy is used successfully more and more in various fields like biology, physics, chemistry, medicine, etc. [10,11,12].

Various spectroscopic instruments can be used for identifying a wide range of gaseous molecules. All the spectroscopic methods give qualitative and quantitative data information by measuring the light absorption in a substance, directly or through a side effect.

Infrared absorption spectroscopy is a common method to acquire the spectral signatures of molecules with high resolution and high sensitivity, allowing detection of ethylene at sub-ppb levels with a partial pressure of 10^−10^ atm and a lowest measurable concentration of 0.9 ppb [12,13,14,15]. Thus, in recent years, a greater accent and interest were given to the photoacoustic spectroscopy laser-based instrument due to its higher sensibility with respect to the traditional spectroscopic techniques.

The aim of this research was to use laser-based photoacoustic spectroscopy technique to evaluate the effect of Pb on common wheat (*Triticum aestivum*) plantlets, by monitoring the ethylene and carbon dioxide (as volatile organic compounds) emitted by the plantlets. We also checked the plant’s development, and we evaluated cell root morphology using fluorescence microscopy. All data showed that lead affects both the morphology and physiology of the plants.

## 2. Results

### 2.1. Lead Effects on Plantlets Tissue Respiration

Ethylene and carbon dioxide molecules generated by the plantlet samples were measured at 200 h from the germination of the *Triticum aestivum* seeds in water or in the presence of 2 µM Pb.

**Figure 1:**
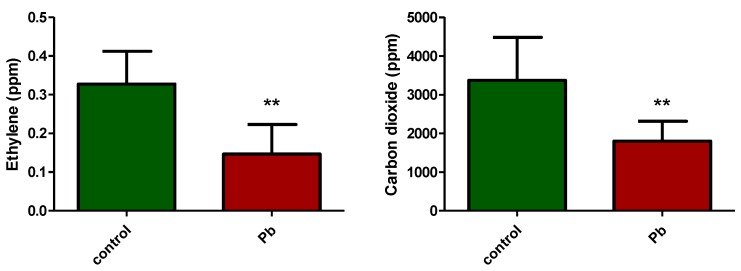
Ethylene and carbon dioxide production for common wheat seeds *(Triticum Aestivum)* germinated in normal (control) conditions or exposed to Pb. * *p* < 0.05, ** *p* < 0.01, *** *p* < 0.001 for unpaired t-test with Welch’s correction.

Figure 1 shows the level of the gases emitted by the *Triticum aestivum* seeds after 200 h of germination with or without Pb.

The concentrations of the two gases measured for the control condition were about 0.33 ± 0.08 ppm for ethylene and 3373.83 ± 1107.00 ppm for carbon dioxide.

Lead intake by *Triticum aestivum* seeds affected both the growth of the plant as well as the concentration of the gases emitted. The ethylene concentration decreased more than half, from 0.33 ppm in the control samples to about 0.15 ± 0.08 ppm for the Pb treated ones. In addition, we found a similar effect on the concentration of CO_2_, which reached about 1802 ± 512 ppm.

The results indicate that exposure of the plants to concentrations around 2 µM Pb affects their respiration significantly.

### 2.2. Lead Phytotoxicity

**Figure 2:**
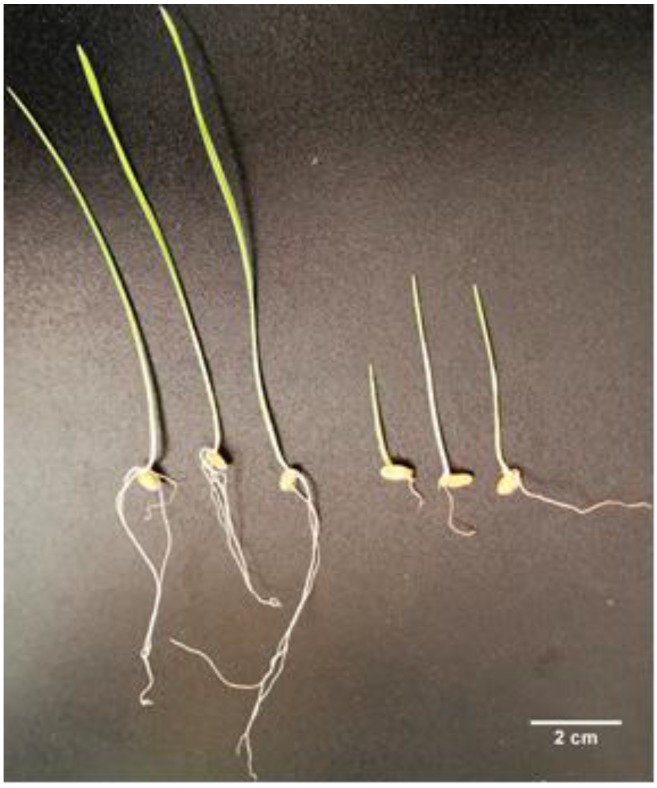
Effect on the growth of common wheat seeds *(Triticum Aestivum)*. The three plants on the left were grown in the control condition, and the ones on the right in the presence of Pb.

**Figure 3:**
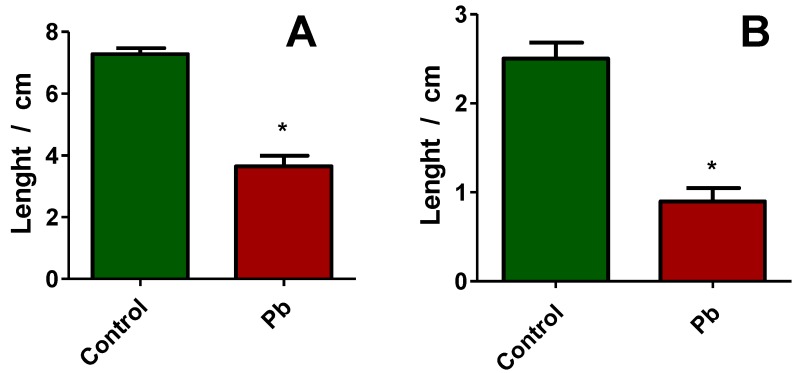
Effect on the development of common wheat seeds *(Triticum Aestivum)* germinated in normal (control) condition or exposed to Pb: (**A**) Stem length and (**B**) roots length * *p* < 0.05, ** *p* < 0.01, *** *p* < 0.001 for unpaired t-test with Welch’s correction.

We further evaluated the effect of Pb on the germination and growth of *Triticum aestivum* plants. Figure 2 presents three representative plants for each growing condition: On the left, the control ones, and on the right, the one treated with 2 µM Pb. One can observe that the seeds grown in the control condition, we have a well-developed plantlet, with long roots and stems. When measured, the average length of the stem was around 7 cm, while the roots were, on average, around 2.5 cm (Figure 3A,B).

When treated with Pb, we see that the germination of the seeds was affected, and both the stems and the roots were significantly smaller compared with the control ones (Figure 2 and Figure 3). The average stem length was 3.8 cm, resulting in a 45% decrease compared to control ones. The average length of the roots, when treated with Pb, was 0.9 cm, resulting in a 64% decrease compared with the control plants.

### 2.3. Lead Effects on the Root Cells Morphology

**Figure 4:**
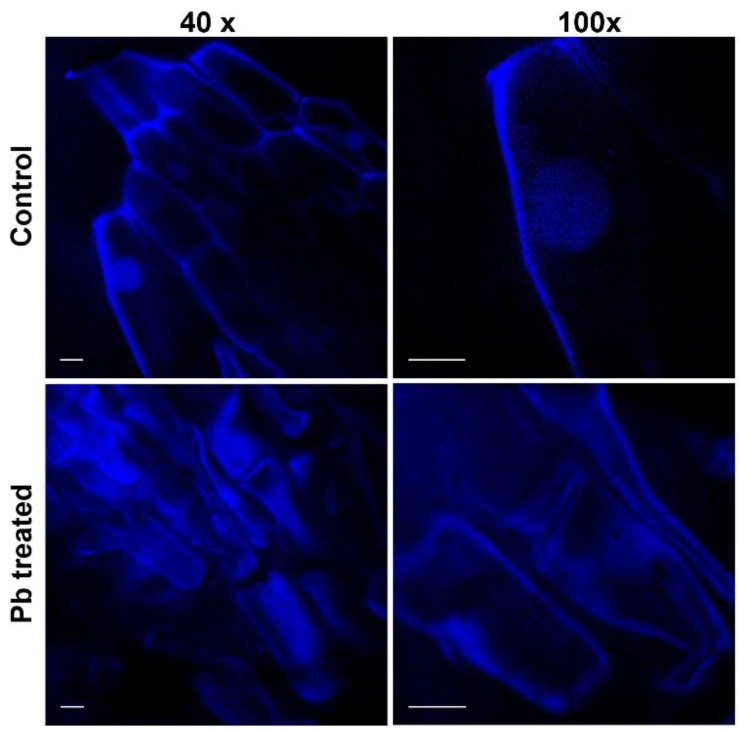
Confocal spinning disk microscope images of the *Triticum Aestivum* root cells exposed or not to Pb and stained with Hoechst 33324. Images were taken with different magnifications (40×, 100×), and the scale bar represents the same value for all images.

Considering the fact that Pb accumulates in high concentrations in roots, we wanted to observe the impact it has on the morphology of the cells in the root, more precisely in the meristem region or in the area of elongation (Figure 4).

We observed that in control roots, the morphology of the cells was unaltered, as we saw a spherical and well-defined nucleus and intact cell walls (see Figure 4, 40× and 100× magnifications).

However, for Pb treated roots, the cells were altered, as they became smaller, with an irregular shape, while the nucleus was reduced and found closer to the cell wall (see Figure 4, 40× and 100× magnification).

## 3. Discussion

Due to various anthropogenic activities, heavy metals, and especially lead had become a serious problem for the environment and leaving organisms [16]. A number of studies have shown [2,3,4,5,17,18,19,20,21] that the toxicity and carcinogenicity of heavy metals found in high amounts, is caused by reactive oxygen species generation and oxidative stress. Because Pb can accumulate in the soil easily, it becomes a source of pollution in the first place for essential crops like: Rice, corn, wheat, etc. Lead is also known to affect enzyme activities, which disrupt important metabolic processes like: Plant growth, respiration, photosynthesis, ion transport, adenosine triphosphate (ATP) content, etc. [22,23]. One of the molecules that is tightly tied to plant development and growth during its entire life, is ethylene [24]. Thus, changes in the ethylene signaling transduction pathway alter the concentration of ethylene, which in the end leads to growth impairment. Because plant development is tightly correlated to plant respiration and photosynthesis, disruption of the ethylene pathway can also affect the Krebs and Calvin cycles, thus altering the concentration of H_2_O, CO_2,_ or other metabolites. These elements can then be transferred through diffusion and released into the respiration of plantlets.

In order to find an easier and faster method to assess markers that can highlight Pb impact on the plant’s respiratory activity, which can further be linked to a deficiency in different physiological processes; we used in this study CO_2_ laser-based photoacoustic spectroscopy. Using this technique, we could compare the gases found in the plant tissue respiration when seeds were treated or not with Pb.

This study follows the effects induced by Pb on the *Triticum aestivum* seeds during its germination and growth by monitoring both the respiration of the plants as well as their morphology.

Considering the above mentioned, we chose to monitor the changes in the concentration of ethylene and carbon dioxide molecules for this study. Similar to our previous study, we found that lead treatment affected the levels of ethylene and carbon dioxide vapors emitted in the respiration of the plantlets, specifically by decreasing their concentration. Previous studies have shown that a CO_2_ decrease was correlated to stomatal closure [22]. In addition, depending on the stress factor and type of plants, it was reported that decreased levels of ethylene can inhibit plant growth [23].

Previous studies have shown that increasing doses of Pb can cause metabolic disorders and can affect plant germination by altering its development, as well as the seed sprouting [21,25,26,27,28,29,30,31,32,33,34]. Surprisingly, at low concentrations (micromolar range) of lead, an inhibition on germination was observed [35], while at high concentrations, we get an opposite effect to correlate with various adverse effects [28,30,36]. Aside from this, both the development of the roots was more affected as compared with the development of the stem [16,22,23,24,36].

We observed similar effects when checking the plants grown in the presence of 2 µM of lead. Their growth was altered by the Pb presence. Both the stem and the roots were less developed when the seeds were grown in the presence of Pb. Their length was decreased to half as compared with the control plants. This effect was expected considering Pb accumulates in high concentration in the roots of various plants, as compared to other plant segments [10,28,29,30,31,32,33,36]. Previous studies reporting lead effect on roots and stem growth for different plants (wheat, guar, sesame, rice, and *Fagopyrumkashmirianum*), have shown that micromolar concentrations inhibit more the growth of the root as compared with growth of the stem [37,38]. These results correlate with the changes found in the level of the respiration gases of the plants. Finally, images recorded in this study show that the morphology of cells in the root is influenced by Pb contamination, by altering the shape of the cell and as well as the nucleus. Similar results were reported for other harmful metals [10,21,33,34,35,36,37,38,39], but also for plants exposed to Pb [20,35,36,37,38,39]. A more in-depth analysis is needed to better understand how altering the molecular pathways changes both gas levels and plant morphology when exposed to lead or other heavy metals. However, our results showed that the laser photoacoustic spectroscopy can be used to give fast and accurate results and support its use in the future for similar investigations.

## 4. Materials and Method

### 4.1. Growth Condition

For the experimental investigation, we used ~5 g of common wheat seeds for each sample (with a total of 6 samples with treated seeds and 6 control samples with distilled water). The seeds were grown in colorless polycarbonate containers with a specific volume of 0.83 cm³/g [10,13,14] and were carried out at room temperature. In each colorless polycarbonate container, 5 g of seeds were added over which we added a total of 25 mL of distilled water for the control samples, and 10 mL with distilled water with 15 mL of Pb for treated seeds. The solutions were replenished every day.

We used a lead solution (composed of a non-radioactive metal salt concentration of 0.4171 mg/L or 2 µM) obtained from the Research and Development National Institute for Metals and Radioactive Resources, Bucharest, Romania (LTTPM1/2017) [10]. The concentration of the lead solution was determined by using a Flame Atomic Absorption Spectrometric Determination of Trace Amounts of Pb with a 0.001 mg/L, detection limit.

Since Pb is toxic and is stored faster than is metabolized or processed, it was decided to make the experimental evaluation of common wheat plantlets at about 200 h (about 9 days), after the germination of the seeds.

### 4.2. CO_2_ Laser-Based Photoacoustic Spectroscopy Experimental Procedure

In this research study, the applicability of CO_2_ laser photoacoustic spectroscopy in the evaluation of common wheat plant respiration was investigated.

**Figure 5:**
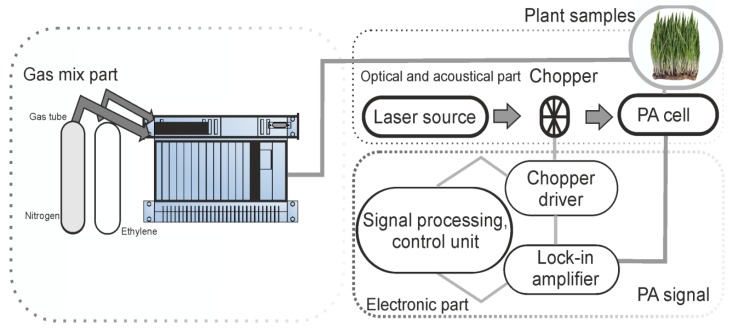
The CO_2_ laser-based photoacoustic spectroscopy system for common wheat plantlets evaluation.

Our experimental research involved the assessment of ethylene and carbon dioxide molecules using the CO_2_ laser photoacoustic spectroscopy, which is graphically illustrated in Figure 5 and fully discussed in other research manuscripts [21].

Summarily, the photoacoustic detection system, based on the resonant photoacoustic detector cell (containing the gas mixture sample) and a tunable continuously wave CO_2_ laser source as the source, had been used. The detection route was followed by a gas mixture system constructed for proper control of the gas molecules under the research study. The gas handling system mad secure the gas purity in the photoacoustic cavity, and it could be used to pump out the cavity, to fix up the common wheat plantlet samples in the cavity, monitor the total and partial pressures of the gas mixture, and also can achieve several actions without making necessary any detachments [40].

To measure multiple gas concentrations, it is necessary to adjust the cavity cell with a known gas mixture and to demonstrate the linearity of the detector signal with the concentration of the probed gas over orders of magnitude. The linear responses of the photoacoustic cavity for low detection limits of ethylene and carbon dioxide were presented substantially in [15,21,40] and were experimentally determined using commercially prepared, certified gas mixtures diluted in pure nitrogen.

The value of the acoustic signal in the photoacoustic spectroscopy measured by the microphones, normalized to the magnitude of the CO_2_ laser radiation power, was proportional to the molecular absorption coefficient of the analyzed common wheat plantlet samples at the CO_2_ laser radiation wavelength used. The CO_2_ laser was kept tuned at absorption line peaks of ethylene at 10.53 μm and carbon dioxide at 9.53 μm [41,42].

Even older than 60 years, this absorption technique has actually seen remarkable development due to the great number of applications for real-time non-invasive analysis of gaseous molecules, especially in the fields of medicine, biology, and environmental pollution monitoring.

The laser spectroscopy apparatus that we used has already produced results in the fields of biology and medicine and yielded the detection of trace gases both in vitro and in vivo [15,21,40,41,42,43,44].

For the photoacoustic response of ethylene and carbon dioxide molecules, the following parameters (Table 1) were used throughout the experiments for common wheat plantlet gas detection.

To evaluate the plantlets tissue signal response from the polycarbonate container (that enters in a small glass cuvette connected to the photoacoustic cavity), we removed the extra gas, and then we flushed the system with pure nitrogen at atmospheric pressure for few minutes. After the system was flushed, we transferred the gas from the sample by using a synthetic airflow near atmospheric pressure.

### 4.3. Evaluation of Lead Phytotoxicity

*Triticum aestivum* seeds were grown in tubes containing 1% agarose gel, in the presence or absence of 2 µM Pb, for 200 h, at room temperature. In the end, the plants were imaged, and the length of the roots and plantlet were measured using ImageJ software (NIH, Bethesda, MD, USA, 2016).

### 4.4. Root Staining and Fluorescence Imaging Experimental Procedure

*Triticum aestivum* roots, untreated and Pb treated, were imaged using a DSD2 confocal spinning disk (Andor, Belfast, UK) mounted on an upright microscope, B51 (Olympus, Hamburg, Germany) equipped with a 40× and 100× objective (Olympus, Hamburg, Germany). The roots were dyed with Hoechst 33324 (Thermo Fisher Scientific, Dreieich, Germany) as described previously [10]. Briefly, the roots were washed 3 times in PBS (phosphate buffer saline), fixed in methanol overnight, and stained for 1 h with 18 µg/mL Hoechst 33324. Finally, the roots were washed, mounted on a glass slide, and images were recorded using the DAPI filter cube (excitation filter 390/40 nm, dichroic filter of 405 nm and emission filter 452/45 nm) from the microscope.

### 4.5. Statistical Analysis

All data were presented as mean ± standard deviation if not stated otherwise, from at least 4 independent experiments. The statistical analysis was performed using GraphPad Prism 5 software (GraphPad Software, Inc., San Diego, CA, USA, 2010) and applying the unpaired t-test with Welch’s correction. *p* < 0.05 was considered statistically significant.

## 5. Conclusions

In the current study, we analyzed the ethylene and carbon dioxide gas molecules using CO_2_ laser-based photoacoustic spectroscopy for seeds germinated with Pb and compared it with the ones recorded for control plantlets. Lead treatment affects ethylene and carbon dioxide vapors in the respiration of plantlets, by largely decreasing their concentration. Both roots and stem lengths are reduced when the seeds are grown in the presence of Pb. Fluorescence microscopy of the *Triticum aestivum* roots strengthened our findings, showing that Pb treatment affects the morphology of the cells found in the meristematic region. Based on our findings, the laser photoacoustic spectroscopy is an easy and accurate technique, which can be used to assess the changes in molecular markers levels of plants found exposed to Pb or other stressors.

## Figures and Tables

**Table 1:** Set-up parameters used for ethylene and carbon dioxide molecules detection.

Work Parameters	Specifications
The total amount of seeds used for germinations	≈5 g
Plant sample pressure	≈1014 mb
Total amount of Pb used for germination	15 mL
Total amount of distilled water used for germination	25 mL
CO_2_ laser line for ethylene detection	10P(14); λ = 10.53 μm; α = 30.4 cm^−1^ atm^−1^
CO_2_ laser line for carbon dioxide detection	9P(18); λ = 9.533 μm; α = 0.00301 cm^−1^ atm^−1^
Synthetic air flow	Linde gas: 20% oxygen, 80% nitrogen (impurities: Hydrocarbons max. 0.1 ppmV, nitrogen oxides max. 0.1 ppmV)
Nitrogen flow	Linde gas 6.0, purity 99.9999%
Working temperature	≈23–25 °C
Polycarbonate containers total volume	0.83 cm^3^/g
Glass cuvette total volume	150 cm^3^
Photoacoustic total volume	1000 cm^3^
Responsivity of the photoacoustic cell	330 cmV/W
Plant sample time analysis	≈5 min
